# Retrospective Evaluation of the Impact of a Multidisciplinary One-Year Online Lifestyle Intervention on Type 2 Diabetes Remission in Nonobese Indian Patients

**DOI:** 10.1155/jdr/3783469

**Published:** 2025-08-16

**Authors:** Pramod Tripathi, Anagha Vyawahare, Nidhi Kadam, Diptika Tiwari, Baby Sharma, Thejas Kathrikolly, Malhar Ganla, Banshi Saboo

**Affiliations:** ^1^Department of Management, Freedom From Diabetes Clinic and Research Foundation, Pune, Maharashtra, India; ^2^Research Department, Freedom From Diabetes Research Foundation, Pune, Maharashtra, India; ^3^Department of Medicine, Dia Care-Diabetes Hormone Clinic, Ahmedabad, Gujarat, India

## Abstract

**Background and Objective:** Despite the high prevalence of T2D among nonobese Indians, there is a notable lack of comprehensive lifestyle intervention studies that target this population. This retrospective study was aimed at filling this gap by evaluating the impact of a multidisciplinary 1-year online intensive lifestyle intervention (ILI) on T2D remission in nonobese Indian patients.

**Methodology:** Retrospective data from medical records of 1453 nonobese patients (BMI 18.5–24.9 kg/m^2^) (aged > 18 years) who participated in a 1-year online subscription-based ILI program at the Freedom from Diabetes Clinic, India, between June 2020 and October 2023 were extracted for analysis. The program included a plant-based diet, physical activity regimens, psychological support, and medical management. Homeostatic model assessment of insulin resistance (HOMA2-IR) and beta cell function (HOMA2%B) were calculated. Remission was defined as maintaining glycated hemoglobin (HbA1C) < 6.5%, measured at least 3 months after cessation of glucose-lowering pharmacotherapy.

**Results:** The study included 1453 patients (72% male), with a median age of 53 years (IQR: 15), BMI of 23 kg/m^2^ (IQR: 2.2), and diabetes duration of 10.5 years (IQR: 10.4). Postintervention, significant changes were observed, including a reduction in weight (−1.5 kg), fasting blood glucose (−13.2 mg/dL), fasting insulin (−0.4 *μ*U/mL), HOMA2-IR (−0.07), and HbA1c (−1.2%), along with an increase in HOMA2%B (+3.1) (*p* < 0.05). Remission was observed in 24% of the patients. The study identified the baseline predictors of remission as being over 40 years of age at onset, having a diabetes duration of less than 6 years, maintaining good glycemic control (HbA1C ≤ 7%), and being drug-naïve. Postintervention factors, such as weight loss and improved beta cell function, were also significant predictors of remission (*p* < 0.05).

**Conclusion:** These findings suggest that a multidisciplinary lifestyle intervention program can significantly improve glycemic control and promote T2D remission in nonobese Indian patients in a real-world setting, thereby highlighting the importance of early intervention and weight management in this population.

**Trial Registration:** Clinical Trials Registry of India identifier: CTRI/2024/03/064596

## 1. Introduction

The prevalence of Type 2 diabetes (T2D) in India is rising, largely due to the global obesity epidemic affecting both developed and developing countries [1]. Despite the worldwide focus on obesity as a major driver of T2D, it is reported that 60%–80% of T2D patients across Asia are nonobese [2]. These individuals account for approximately 80% of India's T2D population and present unique challenges and treatment needs [2, 3] as a result of increased cardiovascular risk due to “metabolic obesity” (high abdominal fat despite a normal body mass index [BMI]) [1]. Additional factors, including significant beta cell dysfunction, minimal insulin resistance, and genetic predispositions, such as markers of beta cell fragility, contribute to a distinct phenotype [4, 5]. As demonstrated in previous research, beta cell dysfunction is the primary etiological factor in the development of T2D among nonobese patients, in contrast to the predominant role of insulin resistance observed in obese individuals [2]. Despite these distinct characteristics, research on T2D remission strategies in nonobese Indian patients remains limited, highlighting a significant gap in our understanding of its unique pathophysiology in the Indian context.

Current research and treatment strategies predominantly focus on obesity-driven diabetes and often overlook the distinct causes of nonobese T2D [6, 7]. Furthermore, given the unique challenges faced by these individuals, the effectiveness of lifestyle interventions commonly applied in obese populations [7–12] remains understudied in this key demographic. Weight loss induced by various interventions is the primary focus of T2D remission strategies [7–12]. Although it may still contribute to remission in nonobese individuals due to the prevalence of metabolic obesity [13], evidence of its effectiveness in Indian populations is sparse. For instance, the ReTUNE study demonstrated that > 6% weight loss in nonobese patients (BMI < 27 kg/m^2^) led to diabetes remission [14]. However, as the study population was exclusively white Europeans with higher than normal BMI according to Asia–Pacific cutoffs for obesity [15], its findings may not be applicable to India's genetically and phenotypically distinct populations.

Further, there is a pressing need for studies that explore comprehensive strategies integrating diet, exercise, psychological support, and medical oversight, specifically tailored to the cultural and individual needs of nonobese Indian T2D patients. The importance of customizing these interventions cannot be overstated as they must consider the distinct genetic predispositions, lifestyle habits, and cultural nuances of this population to be successful. Recent studies have shown the potential of lifestyle changes to offer scalable public health solutions for T2D management [8, 10, 16]. However, to maximize their impact among nonobese Indian patients, these interventions must be carefully adapted to address their unique characteristics.

Therefore, the aim of this study was to retrospectively evaluate the efficacy of a 1-year multidisciplinary online ILI in achieving T2D remission among nonobese Indian patients using real-world data to contribute to the underexplored area of nonobesity-related T2D management strategies. Specifically, it sought to identify the impact of such interventions on glycemic control, weight management, and beta cell function.

## 2. Methods

### 2.1. Study Design and Setting

This retrospective study was conducted at the Freedom from Diabetes Clinic in India, which runs a 1-year subscription-based ILI. Data were extracted from the clinical records of all patients who participated in a 1-year online ILI program from June 2020 to October 2023 based on the eligibility criteria.

### 2.2. Eligibility Criteria

Individuals aged over 18 years with a BMI between 18.5 and 24.9 kg/m^2^ and a confirmed diagnosis of T2D either on treatment with oral hypoglycemic agents (OHAs) or with an HbA1C (glycated hemoglobin) ≥ 6.5% without medication (drug-naïve) were included in the study. Additionally, patients were required to have data available for at least three consultations during the 1-year program.

**Figure 1:**
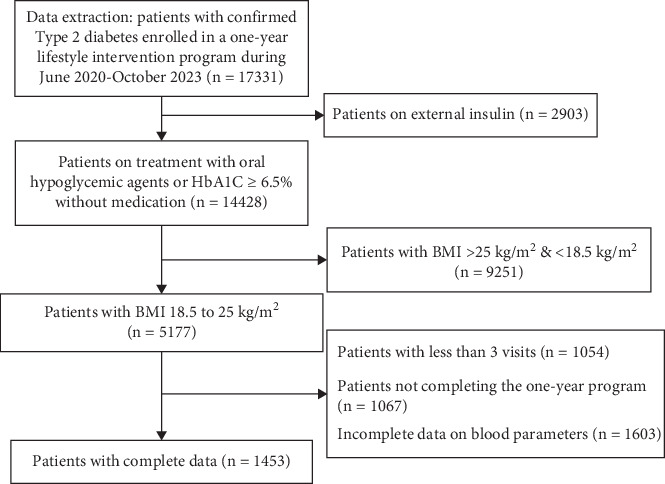
Selection procedure of patients for the study.

Exclusion criteria consisted of other types of diabetes, such as Type 1 diabetes mellitus; diabetes insipidus; maturity-onset diabetes of the young, latent autoimmune diabetes in adults; or gestational diabetes. Participants using exogenous insulin at baseline were excluded from the primary analysis, as insulin therapy alters endogenous insulin levels and would confound the accurate calculation of insulin resistance (HOMA2-IR) and beta cell function (HOMA2%B), which were key metabolic outcomes in this study. Individuals with advanced complications, including nephropathy (eGFR < 30 mL/min/1.73 m^2^, urine microalbumin > 1000 mg), severe retinopathy (nonproliferative and proliferative diabetic retinopathy), and neuropathy (peripheral artery disease, diabetic neuropathy causing numbness, loss of sensation, amputations, ulcers, foot deformities, or Charcot's foot), were also excluded. Additional exclusion criteria included a known history of cancer, pregnancy or lactation, hospitalization for diabetes-related complications within the last 6 months, and incomplete or missing data. The selection procedure for the patient is described in Figure 1.

### 2.3. Ethical Clearance

The study was approved by the Institutional Ethics Committee (Ref. No. FFDRF/IEC/2024/7). The requirement for informed consent was waived by the ethics committee because of the retrospective nature of the study. This study adhered to the ethical principles outlined in the Declaration of Helsinki and its most recent amendments.

### 2.4. Intervention

The 1-year online ILI comprised four integrated protocols: diet, exercise, psychological support, and medical management. The details of the protocol have been described previously [17]. Each patient was assigned a team of six experts: a physician, dietician, physiotherapist, psychologist, mentor, and monitor. The program was delivered through online group sessions and individual consultations. A mobile application enabled communication with experts through calls and messages. As part of the program, each patient owned a glucometer and a digital weighing scale to self-report the data. Participants were enrolled in the intervention for a fixed duration of 12 months, and postintervention measurements were collected at the end of this period to ensure uniform follow-up.

#### 2.4.1. Dietary Intervention

Consisting of a plant-based diet aimed at reducing inflammation through alkalization and detoxification [16, 18], it also involved reducing the overall calorie intake. The details of the protocol have been described previously [17]. For those in the normal BMI category (18.5–22.9 kg/m^2^) [15], overall calorie intake was reduced to 1600 kcal/day with juice fasting advised weekly with meals. Juice fasting, consisting of green smoothie, red, green, and white vegetable juice, was prescribed as an initial short-term intervention to support early weight loss and adherence to dietary transition [19]. Fresh vegetable juices rich in antioxidants and digestive enzymes have been shown to promote metabolic health, improve satiety, and enhance dietary compliance with minimal calorie intake [20]. For those in the overweight category (BMI 23–25 kg/m^2^) [15], the overall calorie intake was reduced to 1200–1400 kcal/day with intermittent fasting in addition to juice fasting. Upon achieving the desired weight loss goals or BMI < 23 kg/m^2^, the dietary focus shifted to a muscle-building regimen and eventually to a maintenance diet (1600–1800 kcal/day) [17]. The muscle-building diet with increased calorie and protein intake in the third phase of the protocol may lead to weight gain (though to a lesser extent than the loss in the first two phases) in the form of muscle mass.

The prescribed dietary intervention followed a structured, whole-food, plant-based pattern. The macronutrient distribution of the prescribed diet was approximately 50%–55% carbohydrates, 20%–25% protein, and 20% fat in the general population, with adjustments for those undergoing a muscle-building diet during later phases where the fats were reduced and protein intake was increased. The dietary intervention was closely monitored by a dietitian in consultation with the assigned physician and physical therapist.

#### 2.4.2. Exercise Routine

The intervention was divided into four phases: Phase 1 focused on improving blood circulation, muscle activation, posture, breathing, flexibility (e.g., yoga), and strength to improve glycemic control; Phase 2 included weight loss, muscle strengthening, and cardiopulmonary endurance while addressing balance and coordination; Phase 3 introduced personalized activities such as swimming, running, and yoga based on the patients' preferences, BMI, and fitness goals; and Phase 4 promoted sustainable fitness through tailored, long-term exercise plans encouraging an independent workout design [17].

#### 2.4.3. Psychological Support

This program aimed to reduce stress and anxiety through group therapy sessions involving meditation, journaling, and positive energy-building activities. Trained psychologists provided individual counseling as needed, using standard therapeutic modalities, such as cognitive behavior therapy and rational emotive behavior therapy [21].

#### 2.4.4. Medical Management

Medical management included daily blood glucose monitoring via a mobile application, nutritional supplementation for deficiencies, and quarterly consultations with an assigned physician. Patients regularly reported their vital signs, including weight, blood glucose levels (BGLs) (fasting and postprandial), and blood pressure, on a mobile application that was monitored by an assigned physician who adjusted the dosage of medications as required [17]. Medication tapering was conducted progressively based on individual clinical responses and daily BGL monitoring through a mobile application. The treating physician reviewed the BGL trends and performed real-time dose adjustments. The tapering approach was guided by the RSSDI-ESI Therapeutic Wheel [22], with sulfonylureas and insulin withdrawn first and metformin or DPP-4 inhibitors continued when appropriate.

Overall, diet, physical activity, and stress management were tailored to each patient's needs, considering comorbidities, age, sex, and fitness level. Although quantitative adherence data were not collected, participant engagement was actively supported through structured follow-up, including weekly or fortnightly consultations with dietitians and physical therapists (later monthly), quarterly physician reviews, and daily self-monitoring of BGLs via a mobile application. On average, each participant received 63 support calls annually. The mobile application enabled continuous interaction through messaging and calls, and the monthly group sessions provided educational and motivational support [17].

### 2.5. Anthropometric and Biochemical Measurements

Baseline sociodemographic data including age and sex were extracted. Data on self-reported anthropometric measurements (height and weight) and biochemical parameters (HbA1C, fasting blood glucose [FBG], and fasting insulin) were extracted from the medical records of the clinic. HOMA2-IR and HOMA2%B were calculated using the homeostatic model assessment (HOMA) calculator [23]. HOMA2‐IR > 2 was considered insulin resistant, and HOMA2%B ≤ 50 was considered low beta cell function [24, 25]. T2D remission was defined as achieving HbA1C < 6.5% measured at least 3 months after discontinuation of all glucose-lowering medications, in line with the international consensus criteria. Medication status was tracked via a mobile application. Remission confirmation was based on HbA1C readings taken at least 3 months after postmedication cessation and within the 12-month program duration. BMI was calculated and categorized based on Asia–Pacific cutoffs [26].

### 2.6. Statistical Analyses

Statistical analyses were performed using IBM SPSS ver. 21 and Python ver. 3.8. Categorical variables are presented as frequencies and percentages, whereas continuous variables are described as mean (standard deviation) or median (interquartile range [IQR]) based on data distribution. The chi-square test was used to test the association between categorical variables. The Wilcoxon signed-rank test was used to test the significance of the difference in parameters (weight, HbA1C, FBG, fasting insulin, HOMA2-IR, and HOMA2%B) pre- and postintervention. The Kruskal–Wallis test was used to test the significance of differences between more than two groups. The absolute percentage change was calculated as the difference between the final and initial values divided by the initial value multiplied by 100. To examine the interplay between diabetes duration, weight loss, and remission, participants were classified using established thresholds from previous studies and the cohort's observed data. A diabetes duration of less than 6 years was selected based on evidence that links shorter disease duration with a higher potential for remission [11, 27–30]. Although the ReTUNE study indicated that a weight reduction of > 6% is associated with remission [14], the mean weight loss in our cohort was 5%; therefore, 5% weight loss was used as the cutoff for stratification. Significant factors from the bivariate analysis were included in the binary logistic regression model (age at diabetes onset, diabetes duration, HbA1C, medicine status at baseline, beta cell function [HOMA2%B], and weight loss postintervention) to assess predictors of remission. Statistical significance was set at *p* < 0.05.

## 4. Results

### 4.1. Baseline Characteristics

Among the 1453 patients, 72% were male, with a median age, BMI, and diabetes duration of 53 years (IQR 15), 23 kg/m^2^ (IQR 2.2), and 10.5 years (IQR 10.4), respectively. At baseline, 62% of the patients were already on a plant-based diet, whereas the remaining 38% transitioned to this dietary pattern as part of the program. Most patients were taking OHAs (93%), while 7% were drug-naïve. Furthermore, 48.5% of the patients were taking statins for dyslipidemia, 33.4% were taking antihypertensive medication, and 13.3% were taking levothyroxine for hypothyroidism. Notably, the prevalence of insulin resistance was low in the study cohort, with 95% of the patients showing no insulin resistance (HOMA2‐IR ≤ 2). At the time of enrollment, 52% (*n* = 760) of the participants were in the overweight category, while 48% (*n* = 693) were in the normal BMI category.

### 4.2. Changes in Biochemical and Anthropometric Parameters Postintervention

Following the intervention, patients showed a significant decrease in weight, HbA1C levels, FBG levels, fasting insulin levels, and HOMA2-IR, with a significant improvement in beta cell function (HOMA2%B) (*p* < 0.05) (Table 1). Additionally, 50.7% (*n* = 385) of the overweight patients shifted to the normal BMI category. The overall remission rate was 24% (27.5% and 15.3% in the weight loss and weight gain groups, respectively). Additionally, 9% of patients were on metformin-only therapy at the end of the intervention, indicating diabetes reversal as defined by Athinarayanan et al. [31]. Patients who shifted from the overweight BMI category to the normal BMI category demonstrated higher remission rates (32.5%) than those who remained in the overweight BMI category (22.1%) and the normal BMI category (19.9%).

### 4.3. Comparison of Baseline Characteristics Based on Remission

The baseline characteristics of the patients were compared between those who achieved remission and those who did not (Table 2). Most of the patients in the “remission” group had a late onset of diabetes (> 40 years of age), were overweight (BMI 23–25 kg/m^2^), had less than 6 years of diabetes duration, had good glycemic control (HbA1C ≤ 7%), were drug-naïve, and showed good beta cell function (HOMA2%B > 50). Patients in the “no remission” group had normal BMI (18.5–22.9 kg/m^2^), showed more than 6 years of diabetes duration, had poor glycemic control (HbA1C > 7%), were on OHAs, and had poor beta cell function (HOMA2%B ≤ 50) (chi-square test, *p* < 0.001).

### 4.4. Effect of Weight Change and Diabetes Duration on Remission

To assess the effect of weight change on remission, patients were divided into five categories: ≤ 5% weight loss, 5%–10% weight loss, > 10% weight loss, weight gain, and no change (Table 3). Of the 1453 patients, 67.3% showed weight loss (median weight loss of 3 kg [IQR 3.6]) while 31.7% showed weight gain (median weight gain of 1.9 kg [IQR 2.3]). Statistically significant association between weight change categories and diabetes remission rates was observed, with higher weight loss being associated with progressively higher remission rates (15.4% for weight gain vs. 35.8% for > 10% weight loss) (chi-square, *p* < 0.001). Similarly, to determine the effect of diabetes duration on remission, patients were divided into two categories (*n* = 1453): diabetes duration > 6 years and ≤ 6 years. Higher remission rates were observed in patients with ≤ 6 years of diabetes (chi-square, *p* < 0.001).

To further assess the combined effect of diabetes duration and weight loss on remission, patients were divided into four groups based on the mean percent weight loss (5%) and the duration of diabetes (6 years) [14] (*n* = 978): Group 1, > 5% weight loss and ≤ 6 years diabetes duration; Group 2, ≤ 5% weight loss and ≤ 6 years diabetes duration; Group 3, ≤ 5% weight loss and > 6 years diabetes duration; and Group 4, > 5% weight loss and > 6 years diabetes duration (Table 4). Higher remission rates were observed in Group 1 (55.1%) and Group 2 (41.6%) than in the other groups (chi-square test, *p* < 0.001). Notably, patients with a diabetes duration > 6 years had lower remission rates, irrespective of weight loss. The highest reduction in HbA1C levels was observed in Group 1, followed by Groups 2, 3, and 4 (Kruskal–Wallis, *p* < 0.001).

### 4.5. Predictors of Remission

**Figure 2:**
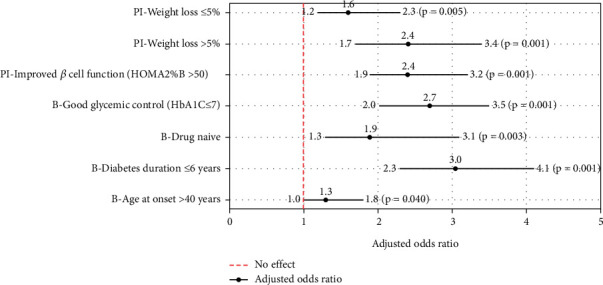
Predictors of remission. The forest plot displays the adjusted odds ratios with 95% confidence intervals (CIs) for various predictors. The adjusted odds ratios are shown as black circles, with the 95% CI indicated by the horizontal black lines. The *p* values are provided in brackets next to the upper CI bound. The vertical dashed red line represents the null value (odds ratio of 1.0), indicating no effect of the predictor. PI, postintervention; B, baseline. Reference category, age at onset ≤ 40 years, diabetes duration > 6 years, on oral hypoglycemic agents, poor glycemic control (HbA1C > 7), Poor beta cell function (HOMA2%B ≤ 50), and weight gain.

All significant factors in the bivariate analysis were included in the regression model (age at diabetes onset, diabetes duration, HbA1C, medicine status at baseline, beta cell function, and weight loss postintervention). Binary logistic regression showed that baseline characteristics such as age > 40 years at diabetes onset, diabetes duration ≤ 6 years, good glycemic control (HbA1C ≤ 7%), and drug-naïve status were significant predictors of T2D remission. Among the postintervention factors, improved beta cell function and weight loss were significant predictors of remission (*p* < 0.05). (Figure 2).

## 5. Discussion

This research showcased real-world data on the effectiveness of a personalized 1-year ILI program in managing T2D in nonobese individuals. Postintervention, 24% of the patients experienced remission along with significant improvements in glycemic parameters (HbA1C, FBG, fasting insulin, and HOMA2%B) and weight. These findings underscore the critical role of dietary modifications, moderate exercise, psychological support, and medical management in T2D patients, which is in contrast to previous studies that primarily focused on diet and medical management, with minimal emphasis on exercise and psychological support for diabetes remission [9–11, 30]. This study represents the first report of remission and management of T2D in a nonobese Indian population through a multidisciplinary 1-year ILI using real-world clinical data.

Previous investigations in obese patients have reported weight loss-driven remission, such as the DiRECT trial reporting 10% weight loss [29], STANDby study in South Asians reporting 10% weight loss [12], and the DIADEM-I trial reporting 12% weight loss [8–12, 14, 29, 30]. Consistent with the findings of the ReTUNE study [14], our research emphasized weight loss as a critical factor in achieving remission, highlighting its importance in the effective management of T2D in the nonobese population. In our study, lower remission rates were observed in nonobese Indian patients. The ReTUNE study reported higher remission rates in nonobese patients (70%) with an initial weight loss of > 6.5% (range 5.5%–10.2%) [14], in contrast to our study's emphasis on > 5% weight loss being strongly associated with remission in the Indian population. In the present study, weight loss of up to 5% was associated with a 1.7-fold higher remission rate, whereas > 5% weight loss was significantly associated with a 2.4-fold higher remission rate. Additionally, 31.7% of patients in our study experienced a median weight gain of 1.9 kg, yet 15% of them achieved remission. This phenomenon may be attributed to an increase in muscle mass during the muscle building phase of the protocol. The remission may be attributable to the visceral fat loss experienced during the first two phases of the diet protocol, which is the basis of remission reported in the ReTUNE trial [14].

This study highlights the role of poor beta cell function in diabetes [5, 32]. Contrary to the prevalent understanding of insulin resistance as the primary factor, 95% of the patients in our study demonstrated high insulin sensitivity while still having diabetes. In contrast, 69% of the patients exhibited poor beta cell function, underscoring its possible role in T2D development. Postintervention, individuals with good beta cell function experienced a 2.4-fold increase in remission rate. Furthermore, in our study, those achieving remission showed a significant improvement in beta cell function, highlighting the importance of multidisciplinary ILI in treating patients with T2D by reducing beta cell workload [3]. Notably, a substantial number of patients who gained weight also achieved remission, which may be attributed to the improvement in beta cell function despite the postintervention weight gain.

Baseline factors such as later age at onset (> 40 years), shorter diabetes duration, good glycemic control at baseline (HbA1C ≤ 7%), and medication status were identified as predictors of remission, emphasizing the potential benefits of addressing these factors early in the disease trajectory. A significant finding was the strong association between remission, good glycemic control, and shorter diabetes duration. Patients with diabetes duration of ≤ 6 years achieved higher remission, irrespective of weight loss (> 5% or ≤ 5%). Recent studies in patients with T2D and obesity have also reported higher remission rates in patients with shorter diabetes durations [11, 27–30], emphasizing the importance of early intervention and lifestyle modifications to achieve remission. The patients in the DiRECT, STANDby, and ReTUNE study had shorter diabetes duration (≤ 6 years) than our study patients (10.5 years) [12, 14, 29] which could explain the higher remission rates observed in these studies as compared to ours. While some findings align with those of the ReTUNE trial (i.e., weight loss), this study uniquely identified shorter diabetes duration and good glycemic control as novel predictors of T2D remission in nonobese patients with T2D in India [14].

The present study, based on data from 460 cities in India, is limited by its retrospective design, self-reported data, lack of a control group, and short 1-year duration, which restricts the ability to attribute changes solely to the intervention or assess long-term diabetes remission. Furthermore, the subscription-based model may have limited access to individuals with a higher socioeconomic status and better educational levels, which may have contributed to greater adherence and potentially influenced the observed remission rates. Selection bias is also a possibility, as patients enrolled in the program may generally be self-motivated with better access to resources for lifestyle changes. Furthermore, participants who dropped out or had incomplete follow-up data were excluded from the final analysis, which followed a per-protocol approach. This may introduce a selection bias and limit the generalizability of the findings. Despite the strengths of our structured intervention, another key limitation is the absence of objective, quantitative adherence data for components, such as diet, physical activity, and behavioral engagement. While adherence was encouraged and supported through regular consultations and mobile app interactions, reliance on self-reported data may have introduced recall or reporting bias. Further, the online format may have impacted adherence due to factors such as technology access and participant engagement; however, we attempted to mitigate these challenges through technological support, regular reminders, monthly calls, feedback forms, live sessions, and personalized coaching. Future studies should incorporate validated adherence measures to better understand the relationship between intervention fidelity and clinical outcomes. Despite these limitations, our findings are based on real-world data that are clinically significant and underscore the potential for T2D remission in nonobese populations.

In conclusion, the 1-year lifestyle intervention program significantly improved glycemic parameters and promoted T2D remission in the nonobese Indian population. This study highlights the critical role of beta cell function and demonstrates the efficacy of early intervention and weight management in achieving diabetes remission. These findings support the integration of a multidisciplinary approach to ILI in the treatment regimen for nonobese T2D patients, suggesting a paradigm shift in diabetes management. Further randomized controlled trials with longer follow-up periods are warranted to investigate the long-term sustainability of remission and the potential of integrating technology-based interventions with traditional healthcare systems.

## Figures and Tables

**Table 1:** Changes in anthropometric and biochemical parameter postintervention (*N* = 1453).

**Parameters**	**Baseline**	**Postintervention**	**Mean difference (95% CI)**	**p** ** value**
Weight (kg)				
Mean ± SD	64 ± 7.7	62.3 ± 7.9	−1.48 (−1.66 to −1.31)	< 0.001
Median (IQR)	64.2 (10)	63 (10.1)		
HbA1C (%)				
Mean ± SD	7.8 ± 1.8	6.6 ± 0.9	−1.23 (−1.31 to −1.14)	< 0.001
Median (IQR)	7.4 (2.1)	6.5 (1.1)		
Fasting blood glucose (mg/dL)				
Mean ± SD	140.4 ± 48.9	127.2 ± 31.02	−13.15 (−15.63 to −10.67)	< 0.001
Median (IQR)	129 (47.8)	122.2 (34.3)		
Fasting insulin (*μ*U/mL)				
Mean ± SD	7.6 ± 6.5	7.3 ± 5.4	−0.35 (−0.67 to −0.04)	0.025
Median (IQR)	6.3 (5.6)	6.1 (4.8)		
HOMA2-IR				
Mean ± SD	0.95 ± 0.8	0.87 ± 0.7	−0.07 (−0.12 to −0.03)	0.001
Median (IQR)	0.8 (0.7)	0.7 (0.6)		
HOMA2%B				
Mean ± SD	42.8 ± 29.7	45.9 ± 30.1	3.08 (1.46–4.70)	< 0.001
Median (IQR)	36.3 (32)	39.8 (29.9)		

*Note:* All values for baseline and postintervention are reported as both *m*ean ± SD and median (IQR). Pre- and postintervention measurements were compared by Wilcoxon's signed-rank test. Intervention effects reported as mean differences for all parameters.

Abbreviations: CI, confidence interval; HbA1C, glycated hemoglobin; HOMA2%B, homeostatic model assessment of beta cell function; HOMA2-IR, homeostatic model assessment of insulin resistance; IQR, interquartile range; SD, standard deviation.

**Table 2:** Comparison of baseline characteristics based on remission.

**Parameter**	**Remission (** **N** = 343**)**	**No remission (** **N** = 1110**)**	**Odds ratio**	**95% CI**	**p** ** value**
Age at onset, *N* (%)			1.73	1.34–2.23	< 0.001
> 40 years	227 (66.2%)	588 (53%)			
≤ 40 years	116 (33.8%)	522 (47%)			
Sex, *N* (%)			1.28	0.96–1.70	0.088
Male	265 (77.3%)	806 (72.6%)			
Female	78 (22.7%)	304 (27.4%)			
Diabetes duration, *N* (%)			0.27	0.21–0.35	< 0.001
> 6 years	180 (52.5%)	889 (80.1%)			
≤ 6 years	163 (47.5%)	221 (19.9%)			
Medicine status, *N* (%)			0.33	0.23–0.48	< 0.001
On OHA	286 (83.4%)	1041 (93.8%)			
Drug-naïve	57 (16.6%)	69 (6.2%)			
Juice fasting, *N* (%)			1.08	0.85–1.38	0.269
No	156 (45.5%)	528 (47.6%)			
Yes	187 (54.5%)	582 (52.4%)			
BMI, median (IQR) and *N* (%)					
Median (IQR)	23.4 (1.9)	22.9 (2.3)	—	—	< 0.001
Normal (18.5–22.9 kg/m^2^)	136 (39.7%)	557 (50.2%)	1.53	1.19–1.96	
Overweight (23–24.9 kg/m^2^)	207 (60.3%)	553 (49.8%)			
HOMA2%B, median (IQR) and *N* (%)					
Median (IQR)	46.8 (38)	34.1 (29.9)	—	—	< 0.001
Poor beta cell function (≤ 50)	183 (53.4%)	820 (73.9%)	0.40	0.31–0.52	
Good beta cell function (> 50)	160 (46.6%)	290 (26.1%)			
HOMA2-IR, median (IQR) and *N* (%)					
Median (IQR)	0.8 (0.8)	0.8 (0.7)	—	—	0.154
Insulin resistance (> 2)	22 (6.4%)	50 (4.5%)	0.68	0.41–1.15	
No insulin resistance (≤ 2)	321 (93.6%)	1060 (95.5%)			
HbA1C, median (IQR) and *N* (%)					
Median (IQR)	6.8% (1.6)	7.6% (2.2)	—	—	< 0.001
Poor glycemic control (> 7%)	144 (42%)	725 (65.3%)	0.38	0.30–0.49	
Good glycemic control (≤ 7%)	199 (58%)	385 (34.7%)			

*Note:* Data are presented as frequency and percentage for categorical variable and as median (IQR) and frequency (percentage) for continuous variable. Odds ratios, 95% CI, and *p* value reported using chi-square test.

Abbreviations: BMI, body mass index; CI, confidence interval; HbA1C, glycated hemoglobin; HOMA2%B, homeostatic model assessment of beta cell function; HOMA2-IR, homeostatic model assessment of insulin resistance; IQR, interquartile range; OHA, oral hypoglycemic agent.

**Table 3:** Remission based on weight loss and diabetes duration.

**Groups**	**Categories**	**Frequency (%)**	**Remission rate**	**p** ** value**
Weight change categories	≤ 5% weight loss	545 (38%)	139 (25.5%)	< 0.001
	5%–10% weight loss	338 (23%)	96 (28.4%)	
	> 10% weight loss	95 (7%)	34 (35.8%)	
	Weight gain	460 (31.7%)	71 (15.4%)	
	No change	15 (1%)	3 (20%)	

Diabetes duration categories	> 6 years diabetes duration	1069 (73.6%)	180 (16.8%)	< 0.001
	≤ 6 years diabetes duration	384 (26.4%)	163 (42.4%)	

*Note:* Data are presented as frequency and percentage; *p* value reported using chi-square test.

**Table 4:** Patient outcomes by weight loss and diabetes duration: Frequency, remission rates, and HbA1C changes.

**Groups**	**Classification based on weight loss and duration**	**Frequency (%)**	**Remission rate**	**HbA1C (% change)**
Group 1	> 5% weight loss and ≤ 6 years diabetes duration	118 (12.1%)	65 (55.1%)	−17.6% (25.1)
Group 2	≤ 5% weight loss and ≤ 6 years diabetes duration	149 (15.2%)	62 (41.6%)	−13.7% (22.5)
Group 3	≤ 5% weight loss and > 6 years diabetes duration	323 (33%)	69 (21.4%)	−11.4% (19.8)
Group 4	> 5% weight loss and > 6 years diabetes duration	388 (39.7%)	73 (18.8%)	−11.1% (17.6)

*Note:* Data are presented as frequency and percentage; data for percentage HbA1C (glycated hemoglobin) change are presented as median and interquartile range.

## Data Availability

Data are available upon reasonable request from the corresponding author.

## References

[1] Vaag A., Lund S. S. (2007). Non-Obese Patients With Type 2 Diabetes and Prediabetic Subjects: Distinct Phenotypes Requiring Special Diabetes Treatment and (or) Prevention?. *Applied Physiology, Nutrition, and Metabolism*.

[2] Olaogun I., Farag M., Hamid P. (2020). The Pathophysiology of Type 2 Diabetes Mellitus in Non-Obese Individuals: An Overview of the Current Understanding. *Cureus*.

[3] Das S., Fonseca V. (2009). Low Bodyweight Type 2 Diabetes in India: Clinical Characteristics and Pathophysiology. *Diabetes & Metabolic Syndrome: Clinical Research & Reviews*.

[4] Carnethon M. R., de Chavez P. J., Biggs M. L. (2012). Association of Weight Status With Mortality in Adults With Incident Diabetes. *JAMA*.

[5] Scott R. A., Fall T., Pasko D. (2014). Common Genetic Variants Highlight the Role of Insulin Resistance and Body Fat Distribution in Type 2 Diabetes, Independent of Obesity. *Diabetes*.

[6] Sarathi V., Kolly A., Chaithanya H., Dwarakanath C. (2017). High Rates of Diabetes Reversal in Newly Diagnosed Asian Indian Young Adults With Type 2 Diabetes Mellitus With Intensive Lifestyle Therapy. *Journal of Natural Science, Biology, and Medicine*.

[7] Praveen Raj P., Bhattacharya S., Saravana Kumar S. (2017). Do Bariatric Surgery-Related Type 2 Diabetes Remission Predictors Add Clinical Value? A Study on Asian Indian Obese Diabetics. *Obesity Surgery*.

[8] Ko J. H., Kim T. N. (2022). Type 2 Diabetes Remission With Significant Weight Loss: Definition and Evidence-Based Interventions. *Journal of Obesity & Metabolic Syndrome*.

[9] Hamman R. F., Wing R. R., Edelstein S. L. (2006). Effect of Weight Loss With Lifestyle Intervention on Risk of Diabetes. *Diabetes Care*.

[10] Wing R. R., Lang W., Wadden T. A. (2011). Benefits of Modest Weight Loss in Improving Cardiovascular Risk Factors in Overweight and Obese Individuals With Type 2 Diabetes. *Diabetes Care*.

[11] Taheri S., Zaghloul H., Chagoury O. (2020). Effect of Intensive Lifestyle Intervention on Bodyweight and Glycaemia in Early Type 2 Diabetes (DIADEM-I): An Open-Label, Parallel-Group, Randomised Controlled Trial. *Lancet Diabetes and Endocrinology*.

[12] Sattar N., Welsh P., Leslie W. S. (2023). Dietary Weight-Management for Type 2 Diabetes Remissions in South Asians: The South Asian Diabetes Remission Randomised Trial for Proof-of-Concept and Feasibility (STANDby). *Lancet Regional Health-Southeast Asia*.

[13] Unnikrishnan R., Anjana R. M., Mohan V. (2014). Diabetes in South Asians: Is the Phenotype Different?. *Diabetes*.

[14] Taylor R., Irvine K. M., Barnes A. C. (2022). 218-LB: Remission of Type 2 Diabetes After Weight Loss in “Normal” Weight People—The ReTUNE Study. *Diabetes*.

[15] Aziz N., Kallur S. D., Nirmalan P. K. (2014). Implications of the Revised Consensus Body Mass Indices for Asian Indians on Clinical Obstetric Practice. *Journal of Clinical and Diagnostic Research*.

[16] Storz M. A., Ronco A. L., Hannibal L. (2022). Observational and Clinical Evidence That Plant-Based Nutrition Reduces Dietary Acid Load. *Journal of Nutritional Science*.

[17] Tripathi P., Kadam N., Tiwari D. (2024). The Diabetes Remission in India (DiRemI) Study: Protocol for a Prospective Matched-Control Trial. *PLoS One*.

[18] Obert J., Pearlman M., Obert L., Chapin S. (2017). Popular Weight Loss Strategies: A Review of Four Weight Loss Techniques. *Current Gastroenterology Reports*.

[19] Tripathi P., Hiremath M., Vyawahare A., Dudhbhate A. (2020). Extent of Diabetic Nephropathy Reversal in Type 2 Diabetes Mellitus Patients by Following the Freedom From Diabetes Protocol. *Indian Journal of Public Health Research & Development*.

[20] Henning S. M., Shao P., Lu Q. Y. (2016). Health Effects of 3-Day Fruit and Vegetable Juice Fasting. *Advances in Nutrition*.

[21] Sohrabi F., Sohrabi A., Shams-Alizadeh N., Cayoun B. A. (2022). Managing Type 2 Diabetes and Depression With Mindfulness-Integrated Cognitive Behavior Therapy (MiCBT). *Discover Psychology*.

[22] Hasnani D., Chavda V., Agrawal D. (2022). Validation of RSSDI Therapeutic Wheel With Clinical Experience of Indian Physicians. *International Journal of Diabetes in Developing Countries*.

[23] Levy J. C., Matthews D. R., Hermans M. P. (1998). Correct Homeostasis Model Assessment (HOMA) Evaluation Uses the Computer Program. *Diabetes Care*.

[24] Saha A. (2022). Pancreatic Beta-Cell Function and Degree of Insulin Resistance Among Newly Detected Type 2 Diabetics and Their Correlation With Anthropometric, Glucose, and Lipid Parameters: An Observational Cross-Sectional Study. *Asian Journal of Medical Sciences*.

[25] Tripathi P., Vyawahare A., Kadam N. (2024). Comparison of Clustering and Phenotyping Approaches for Subclassification of Type 2 Diabetes and Its Association With Remission in Indian Population. *Scientific Reports*.

[26] World Health Organization (2000). *The Asia-Pacific Perspective: Redefining Obesity and Its Treatment*.

[27] Wei J., Chen J., Wei X. (2022). Long-Term Remission of Type 2 Diabetes After Very-Low-Calorie Restriction and Related Predictors. *Frontiers in Endocrinology*.

[28] Karter A. J., Nundy S., Parker M. M., Moffet H. H., Huang E. S. (2014). Incidence of Remission in Adults With Type 2 Diabetes: The Diabetes & Aging Study. *Diabetes Care*.

[29] Thom G., Messow C. M., Leslie W. S. (2021). Predictors of Type 2 Diabetes Remission in the Diabetes Remission Clinical Trial (DiRECT). *Diabetic Medicine*.

[30] Zaghloul H., Chagoury O., Elhadad S. (2020). Clinical and Metabolic Characteristics of the Diabetes Intervention Accentuating Diet and Enhancing Metabolism (DIADEM-I) Randomised Clinical Trial Cohort. *BMJ Open*.

[31] Athinarayanan S. J., Adams R. N., Hallberg S. J. (2019). Long-Term Effects of a Novel Continuous Remote Care Intervention Including Nutritional Ketosis for the Management of Type 2 Diabetes: A 2-Year Non-Randomized Clinical Trial. *Frontiers in Endocrinology*.

[32] Saisho Y. (2015). *β*-Cell Dysfunction: Its Critical Role in Prevention and Management of Type 2 Diabetes. *World Journal of Diabetes*.

